# Target tracking and 3D trajectory acquisition of cabbage butterfly (*P*. *rapae*) based on the KCF-BS algorithm

**DOI:** 10.1038/s41598-018-27520-z

**Published:** 2018-06-25

**Authors:** Yang-yang Guo, Dong-jian He, Cong Liu

**Affiliations:** 1 College of Mechanical and Electronic Engineering, Northwest A&F University, Yangling, Shannxi 712100 China; 2 Key Laboratory of Agricultural Internet of Things, Ministry of Agriculture, Yangling, Shaanxi 712100 China; 3 Shaanxi Key Laboratory of Agricultural Information Perception and Intelligent Service, Yangling, Shaanxi 712100 China

## Abstract

Insect behaviour is an important research topic in plant protection. To study insect behaviour accurately, it is necessary to observe and record their flight trajectory quantitatively and precisely in three dimensions (3D). The goal of this research was to analyse frames extracted from videos using Kernelized Correlation Filters (KCF) and Background Subtraction (BS) (KCF-BS) to plot the 3D trajectory of cabbage butterfly (*P*. *rapae*). Considering the experimental environment with a wind tunnel, a quadrature binocular vision insect video capture system was designed and applied in this study. The KCF-BS algorithm was used to track the butterfly in video frames and obtain coordinates of the target centroid in two videos. Finally the 3D trajectory was calculated according to the matching relationship in the corresponding frames of two angles in the video. To verify the validity of the KCF-BS algorithm, Compressive Tracking (CT) and Spatio-Temporal Context Learning (STC) algorithms were performed. The results revealed that the KCF-BS tracking algorithm performed more favourably than CT and STC in terms of accuracy and robustness.

## Introduction

Insect behaviour has become an important research direction in the field of plant protection^1^. Behavioural research may inform methods for biological control^2,3^, biological model construction^4,5^ and plant-insect interactions^6,7^. To study the behaviour of flying insects accurately, it is necessary to observe and record their flight trajectory quantitatively and precisely in three dimensions (3D).

Traditional detection methods for insect behaviour depend mainly on direct and manual observation, which are complicated by arbitrary qualification, wasting of human resources and low effectiveness^8^. Recent developments in computer vision have stimulated the application of these techniques to insect tracking^9–11^. Straw *et al*.^12^ used three cameras to three-dimensionally track flying animals and can also track flies and birds, but the use of three cameras increased the difficulty of matching and the amount of 3D coordinate calculations. Okubo *et al*.^13^ obtained images of a group of mosquitoes with a single camera and constructed the 3D trajectory of mosquitoes based on the geometric relationship between the mosquito group and its shadow on a white background. Stowers *et al*.^14^ used the FreemoVR platform to establish a height-aversion assay in mice and studied visuomotor effects in Drosophila and zebrafish. However, this method was not directly suitable for the investigation of behaviours for which stereopsis is important because it rendered visual stimuli in a perspective-correct manner for a single viewpoint. Jantzen and Eisner^15^ implemented Lepidoptera’s 3D trajectory tracking, and Lihoreau *et al*.^16^ obtained the three dimensional foraging flights of bumblebees. However, in these studies, the experimental environment was relatively simple, and the target was obvious.

Automated image-based tracking has been applied for outdoor research, and the imaging method includes thermal infrared^17^, sonar^18^, 3D^19^, and harmonic radar^20^ methods. Xu *et al*.^21^ proposed a method for the 3D observation of fish based on a single camera. A waterproof mirror was installed above an experimental fish tank to simulate a camera shooting from top to bottom. Although monocular vision was able to determine a 3D trajectory, the shooting video was largely influenced by environmental factors, and the calculation process was complex. Hardie and Powell^22^ investigated the use of two or more parallel cameras to obtain an image sequence of aphid-landing outdoors, and drew the 3D landing trajectory, but this method involved a complex 3D matching process due to the complicated external environment, which increased the difficulty of matching. Risse *et al*.^23^ used a freely moving camera to track small animals in cluttered natural environments. Animals were represented by a single point, and the animal had to move in the majority of the frames to be tracked correctly. Although outdoor research has scored some achievements, complex natural environments (e.g., light, temperature, spatial structure of habitats, leaves, animals) increases the difficulty of outdoor research. Indoor research can weaken the impact of environmental factors and be conducive to the scholarly understanding of insect behaviour and also provide a technical and theoretical basis for outdoor experiments.

The cabbage butterfly is one of the most harmful insects to agriculture^24^, and thus it is necessary to track them and obtain their flight trajectories. In this study, we chose the cabbage butterfly adult as the research object, and performed research in a wind tunnel to which plants and wind were added to introduce a more natural environment than an empty tunnel. We then designed a orthogonal binocular vision measurement system with top-view and side-view cameras aimed at the wind-tunnel experimental environment, reducing the difficulty of camera matching. The insect can be obscured by plants. We propose a method for tracking and obtaining 3D trajectories based on a combination of the kernelized correlation filter (KCF) algorithm and background subtraction (BS) (KCF-BS). This method can occlusion and re-track the target after the target was lost. The proposed method will provide a theoretical foundation and valuable reference for further behavioural research on this insect and a technical reference for target tracking based on orthogonal binocular vision.

## Results

### Kernel function and characteristic parameter optimization

**Figure 1:**
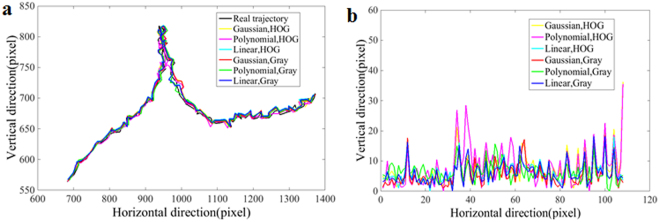
Trajectories using KCF algorithm with different parameters. (**a**) Trajectories with different parameters and real movement. (**b**) Error distance (Euclidean distance) vs. time (frame) for all algorithms and parameters.

To optimize the kernel functions and the characteristics of tracking of cabbage butterfly targets, a top-view video of flight in the wind tunnel was employed as a test. Linear, Polynomial and Gaussian kernels were used successively in tracking tests under Histogram of Oriented Gradient (HOG)^25^ and Gray characteristics, while the actual flight curve of the cabbage butterfly target centroid coordinates in the test video frames was manually marked. Comparisons of the trajectory results from Linear, Polynomial and Gaussian kernels under the two characteristics in the top view video with the actual flight path are shown in Fig. 1a, and the error distance (Euclidean distance) vs. time (frame) for all algorithm and parameters are shown in Fig. 1b. The Euclidean distance in pixels was used to express the relative error between tracking and the actual trajectories, as shown in Table 1.

**Table 1:** Relative position with different parameters (Pixels).

Parameters	Maximum relative error	Minimum relative error	Average relative error
Hog/Gaussian	36.24	1.00	7.12
Hog/Polynomial	35.51	0.00	8.15
Hog/Linear	18.11	0.00	6.59
Gray/Gaussian	18.44	1.00	5.58
Gray/Polynomial	18.25	0.00	6.52
Gray/Ginear	18.25	0.00	6.03

Figure 1a shows that the tracking trajectory and the actual trajectory curves were quite close to each other, but there was a relatively large deviation between the tracking and actual trajectory curves using HOG/Gaussian, and HOG/Polynomials (Fig. 1b) and a relatively smaller tracking error using the Gray characteristic (Fig. 1b and Table 1). Although the average relative error of Gray/Gaussian was smallest, the maximum and minimum relative errors of Gray/Gaussian were larger and might lead to large fluctuations in estimates. Considering this synthetically using multiple factors, Gray/Linear with smaller maximum, minimum and average relative errors was selected as optimal for tracking the 3D trajectory in this paper.

### Target tracking and centroid trajectory acquisition

**Figure 2:**
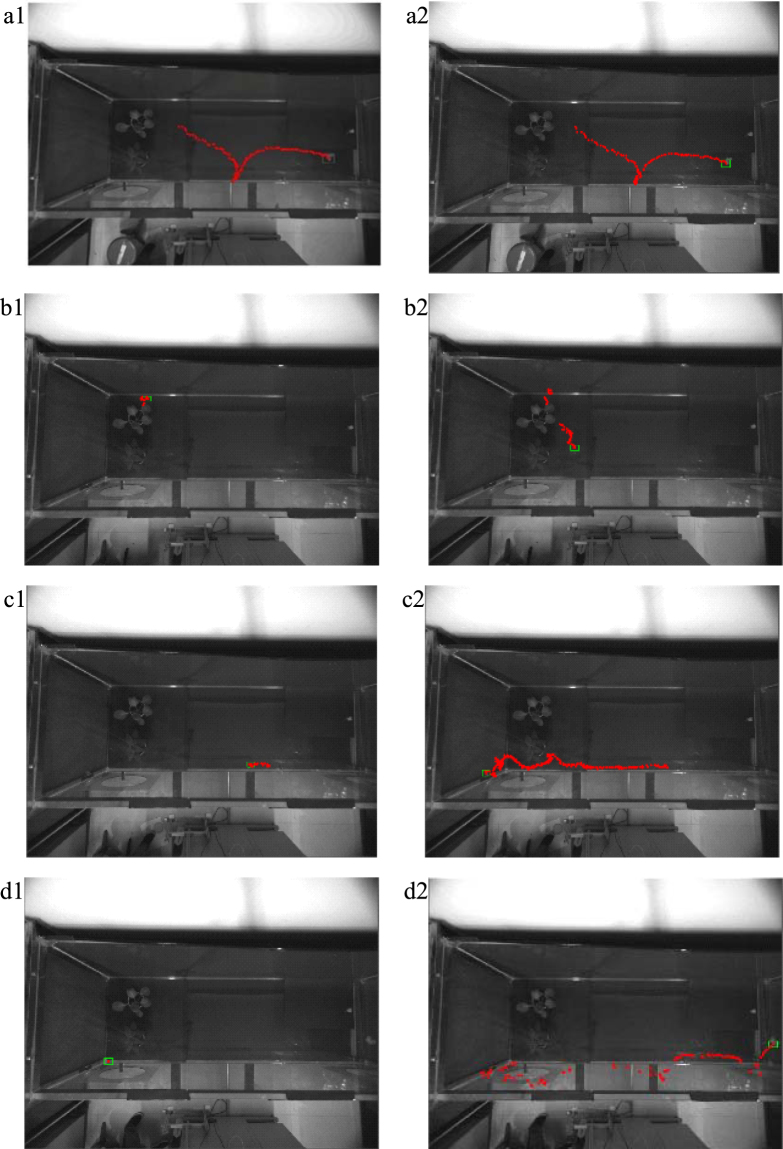
Tracking results of the KCF algorithm (first column) and paper algorithm (second column) in different situations. In all images, the rectangular box is the frame target position at the end of tracking, and the red point is the two-dimensional centroid of the cabbage butterfly. (**a**) Case of target flying in the middle of the wind tunnel. (**b**) Case of obscured target. (**c**) Case of target at edge of wind tunnel. (**d**) Case of similar colours for target and background.

In the preliminary tracking test, the threshold T was 3. The test was conducted in an open area without plant shelter, when the insect was occluded by the plant leaves or was located at the edge of the wind tunnel, and when the background was similar to the target colour. Figure 2 shows tracking data for the insect target in the top-view video based on the KCF algorithm (first column), and the KCF- BS method (second column).

Figure 2a shows the tracking results when the insect was located in the open area in the centre of the wind tunnel, and both algorithms could be used to completely track and plot the trajectory of the target centroid. Figure 2b–d shows the tracking results when the insect was occluded by plant leaves, when located at the edge of wind tunnel, and when the target colour was similar to the background. Comparing the tracking results of the two methods indicates that, in those circumstances, the KCF algorithm alone failed to track continuously but the method of KCF-BS accurately tracked the actual flying trajectory of the cabbage butterfly, but missed information when the background colour was similar to the target. The success rates of the two algorithms in the four cases are shown in Table 2.

**Table 2:** Success rates of KCF and KCF-BS algorithms in four cases in top view video.

Cases	Frames	KCF (%)	KCF-BS (%)
Wind tunnel centre	108	100.0	100.0
Target obscured	58	13.8	81.0
Wind tunnel edge	278	6.1	96.4
Similar colours	196	1.0	52.6

### Comparison experiment

**Figure 3:**
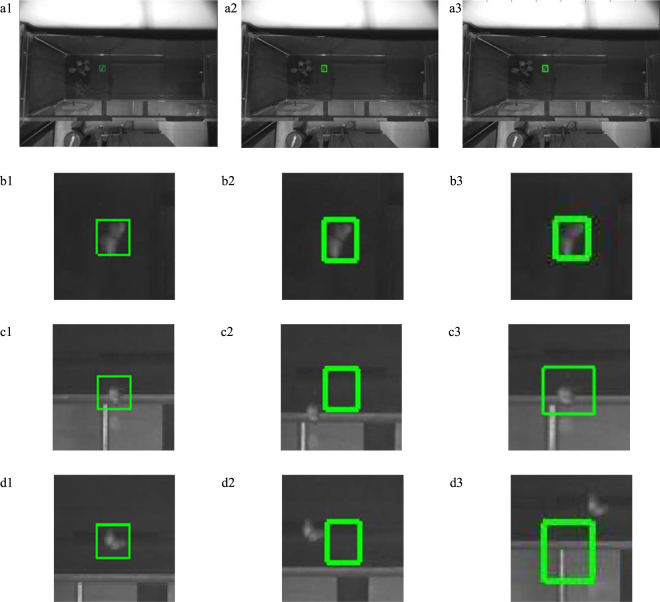
Comparison of objects using the KCF-BS algorithm (first column), CT algorithm (second column) and STC algorithm (third column). In all images, the rectangular box is the frame target tracking position. (**a**) First frame marking results. (**b**) Local map of the first frame. (**c**) Local map of 45th frame in the tracking process. (**d**) Local map of the 59th frame in the tracking process.

To verify the effectiveness of the tracking method described in this paper, Compressive Tracking (CT)^26^ and the Spatio-Temporal Context Learning (STC)^27^ were performed in the top-view video. Figure 3 shows comparisons of the object using the KCF-BS algorithm (first column), the CT algorithm (second column) and the STC algorithm (third column) in the wind tunnel centre case. The CT algorithm dropped the target at the 45th frame (Fig. 3c2) and could not effectively track the target again (Fig. 3d2). The target was lost in the 59th frame using the STC algorithm (Fig. 3d3). The KCF-BS algorithm could still track the target in the 45^th^ and 59^th^ frames.

**Figure 4:**
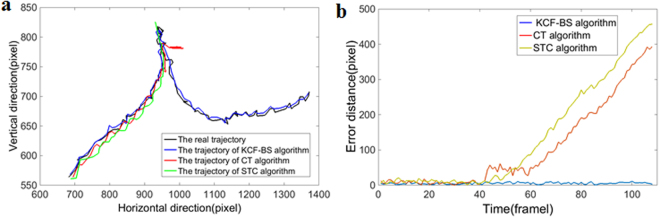
Trajectories using the KCF-BS algorithm, CT algorithm and STC algorithm. (**a**) Trajectories with different algorithms and real movement. (**b**) Error distance (Euclidean distance) vs. time (frame) for all algorithms.

Figure 4a shows the actual trajectory and tracking trajectory using the KCF-BS algorithm, the CT algorithm and the STC algorithm in the wind tunnel centre case, and Fig. 4b shows the error distance (Euclidean distance) vs. time (frame) for all algorithms. CT and STC algorithms could not continuously track the target (Fig. 4a) and showed a large deviation in Fig. 4b. When the target was not lost, the average errors of the CT and STC algorithms were 8.3 pixels and 10.9 pixels, respectively. The KCF-BS algorithm tracked the target continuously with an average error was 7.1 pixels. The success rates of the CT, STC and KCF-BS algorithms in the four cases are shown in Table 3.

**Table 3:** Success rates of CT, STC and KCF-BS algorithms in four cases in top view video.

Cases	Frames	CT (%)	STC (%)	KCF-BS (%)
Wind tunnel centre	108	41.7	54.6	100.0
Target obscured	58	13.8	13.8	81.0
Wind tunnel edge	278	1.1	1.4	96.4
Similar colours	196	2.6	1.5	52.6

### Acquisition of the 3D trajectory

**Figure 5:**
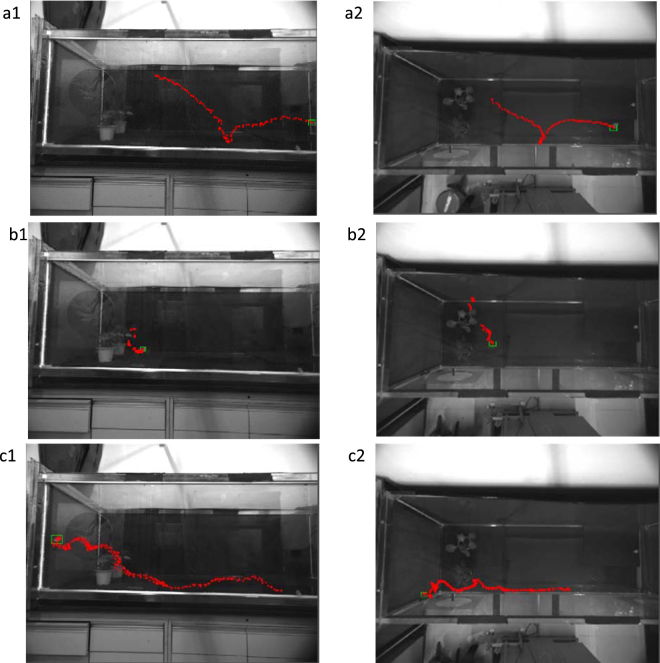
Trajectory results of cabbage butterfly using the herein-presented method. In all images, the rectangular box represents the frame target position at the end of tracking, and the red point is the two-dimensional centroid of the cabbage butterfly. (**a1**) Trajectory results in side-view in the centre of the wind tunnel. (**a2**) Trajectory results in top-view in the middle of the wind tunnel. (**b1**) Trajectory results in side-view in the obscured target case. (**b2**) Trajectory results in top-view for the obscured target situation. (**c1**) Trajectory results in side-view in the target at the edge of the wind tunnel case. (**c2**) Trajectory results in top-view in the target at the edge of the wind tunnel case.

**Figure 6:**
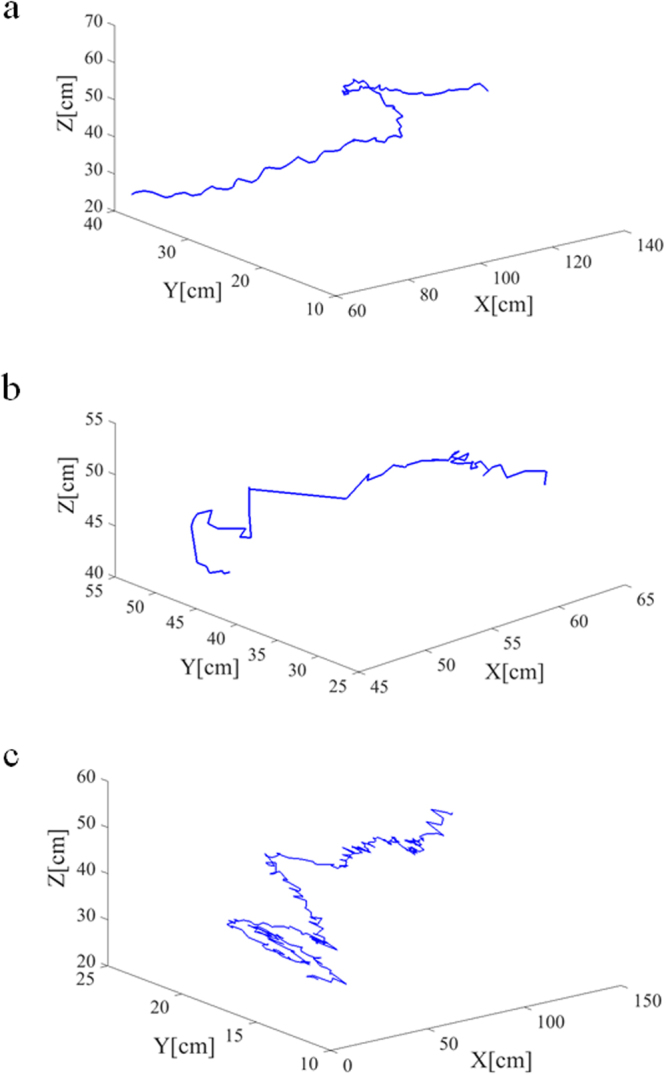
3D trajectory of the moving target. (**a**) 3D trajectory of the moving target in the middle of the wind tunnel. (**b**) 3D trajectory of moving target in the obscured target case. (**c**) 3D trajectory of moving target in the edge of wind tunnel case.

A target-tracking test was performed on the acquired cabbage butterfly video, and a representative result for the side-view and top-view video target tracking is shown in Fig. 5. The 3D spatial coordinate sequence *P*(*x*_*n*_, *y*_*n*_, *z*_*n*_) during cabbage butterfly flying was generated from the coordinate matrices *P*(*x*_*n*_, *z*_*n*_) and *P*(*x*_*n*_, *y*_*n*_) obtained by side-view and top-view video tracking, respectively. Thus, the 3D trajectory of the cabbage butterfly in flight could be drawn (Fig. 6).

Figure 6 shows that the 3D trajectory obtained in the wind tunnel centre case was better and smoother. The abnormality in the 3D trajectory (Fig. 6b) was caused by the occlusion of the plant, as seen from Fig. 5b1,b2, but the target could still be effectively tracked afterwards. In the case of the target at the edge of wind tunnel, the poor 3D trajectory (Fig. 6c) was caused by the similar colour of the background area and the target, which as seen from Fig. 5c1,c2.

### Accuracy evaluation of target three-dimensional coordinates

To evaluate the accuracy of the 3D coordinates of the insect target in this paper, five white ping-pong balls with a diameter of 40 mm were numbered and suspended from fine lines in different locations in the wind tunnel (the fan was stopped), and the actual distances between the ping-pong balls were measured using a steel tape. The 3D spatial coordinates of the centre of each sphere were also obtained using the methods described in this paper, and the Euclidean distance between the balls was calculated. The comparison of the calculated and actual distance between balls is shown in Table 4. The minimum, maximum and average errors were 4 mm, 20 mm, and 8.8 mm, respectively. The standard deviation was 4.9 mm.

**Table 4:** Comparison of calculated and actual distance between balls.

Number	Calculated/mm	Actual value/mm	Error/mm	Relative error/%	Number	Calculated/mm	Actual value/mm	Error/mm	Relative error/%
1–2	301	291	10	3.44	2–4	409	403	6	1.49
1–3	452	445	7	1.57	2–5	511	491	20	2.04
1–4	345	340	5	1.47	3–4	310	302	8	2.65
1–5	275	269	6	1.56	3–5	545	529	16	3.02
2–3	260	256	4	1.56	4–5	266	260	6	2.31

**Figure 7:**
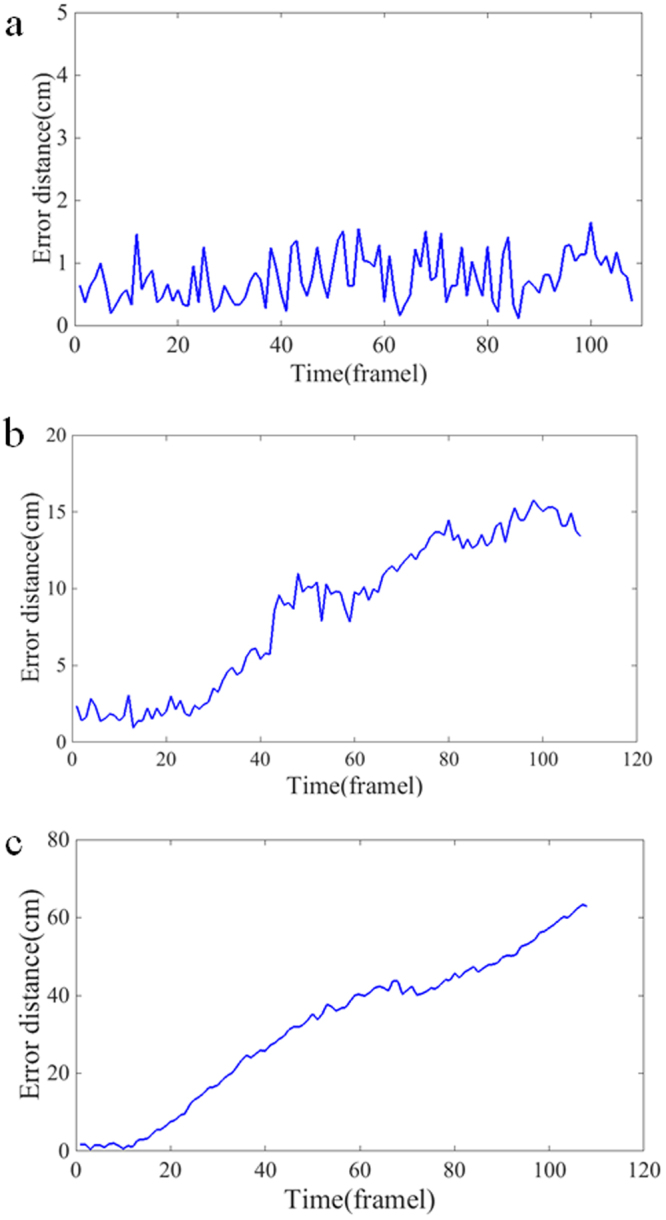
3D trajectory error of actual distance error (cm) vs. time (frame) for three algorithms in the wind tunnel centre case. (**a**) 3D trajectory error of actual distance error (cm) vs. time (frame) for the KCF-BS algorithm. (**b**) 3D trajectory error of actual distance error (cm) vs. time (frame) for the CT algorithm. (**c**) 3D trajectory error of actual distance error (cm) vs. time (frame) for STC algorithm.

The 3D trajectory error of the actual distance of KCF-BS, CT and STC algorithms in the wind tunnel centre case are shown in Fig. 7. The minimum, maximum and average errors of the KCF-BS algorithm were 0.11 cm, 1.65 cm, 0.76 cm, respectively. As seen from Fig. 7b,c, the error of CT and STC algorithms is large because the CT and STC algorithms could not redetect the target when the target was lost, and the position of the tracking box was basically unchanged.

## Discussion

The experiment was performed in the wind tunnel, and cabbage butterfly occupied a small proportion in the scene with deformation, making video capture and tracking detection difficult. To meet the tracking test at 60fps, the present study proposed improved upon KCF and compared these results to KCF.

Tracking experiments using the KCF algorithm found that the algorithm missed the target when the butterfly was occluded by a plant, or when it was near the edge of wind tunnel, or when it was similar in colour to the background due to deformation during flight (Fig. 2b1–d1). For example, when the wings were in the closed state during flying, a partial lack of information led to tracking failure. The grey-black body and white wings of the adult cabbage butterfly resembled the colour of the floor in the top view as seen through the partial area of the glass frame, and thus the target could not be effectively detected. After missing the tracking target, the target box remained in its current position and was unable to re-detect and recognize the target. However, in the above situation, the proposed KCF-BS method could still obtain better test results (Fig. 2b2–d2). The success rates of KFC-BS detection in the cases of wind tunnel centre, obscured target, wind tunnel edge and similar colours were 100%, 81.0%, 96.4% and 52.6%, respectively (Table 2). In the obscured target case, the frames missed by the KCF-BS were the target occlusion frames. In the wind tunnel edge case, the frames lost by KCF-BS were due to the similar colour of the target and the background, as proved in the similar colours case (Fig. 2d2), and KCF-BS missed more target frames, which was a further consideration.

To verify the effectiveness of the tracking method described in this paper, the CT algorithm and STC algorithm were used to assess the performance of the KCF-BS method. As seen from Figs 3 and 4, the KCF-BS method can more precisely track the target in this case.

CT and STC algorithms are commonly used in tracking algorithms^28–31^. The CT algorithm is a discriminant tracking algorithm that uses a rectangular window to represent the tracking result. Most of the pixels in the tracking window come from the target and a small part comes from the background. When sparse local Haar-like features are extracted in the window, the pixels in the background affect the robustness of the algorithm. The cabbage butterfly possess a grey-black body and white wings with fine scales, and has deformation in motion. When the wings of the cabbage butterfly were closed, the background pixels in the tracking window occupy a large proportion, and thus, the CT algorithm could not effectively detect the target. As seen from the second column of Figs 3c2,d2 and 4a, the CT algorithm dropped the target and could not effectively track the target again. This was reflected in Table 3, with a lower accuracy rate. The STC algorithm was based on the generative appearance model and was integrated with tracking, learning and detection. In STC, Zhang *et al*.^27^ added background information including spatial location in the generative appearance model and adopted Fast Fourier Transform (FFT) to reduce calculations, so this algorithm could achieve real-time, robust and efficient tracking results. However, the method was less effective in terms of target distortion, background colour similarity and motion blur. In tracking the cabbage butterfly, the STC algorithm also dropped the target (Figs 3d3 and 4a). This was reflected in Table 3 lower accuracy. When the target was not lost, the average errors of the CT and STC algorithms were 8.3 pixels and 10.9 pixels, respectively. The KCF-BS algorithm could track the target continuously, and the average error was 7.1 pixels. When the target disappeared or the tracking effect was poor, the BS algorithm was used to retrieve the target position. However, when the target was similar in colour to the background, the phenomenon of missing frames was still observed (Fig. 2d2).

The two-dimensional coordinates of the target centroid were obtained by tracking cabbage butterflies in top-view and side-view videos using the KCF-BS algorithm. The 3D coordinates of the target centroid were obtained by matching the corresponding frames in the videos from the two cameras, from which the 3D trajectories was obtained. Comparing the measured distance and actual distance between the balls (40 mm diameter) of the test ball with the Euclidean distance obtained by the algorithm proposed in this paper, the results showed that the average error was 8.8 mm and the standard deviation was 4.9 mm. The distance error was mainly caused by camera calibration error, orthogonal binocular visual matching error and manual measurement error. The calibration pixel errors for the top and side view cameras were (0.51; 0.49) and (0.42; 0.52), respectively. The orthogonal binocular visual matching error, caused by hardware performance, was ignored. The manual measurement errors were inevitable, and we invited experienced researchers to make the measurements to reduce the errors. In Fig. 7, the minimum, maximum and average errors of KCF-BS algorithm were 0.11 cm, 1.65 cm, and 0.76 cm, respectively. The centroid positioning deviation caused by deformation of the cabbage butterfly during flying affected the measurement accuracy of the target. The problem of how to effectively acquire the target centroid must still be solved.

The method in this paper achieves target tracking and obtains three-dimensional motion trajectory, and this study of the cabbage butterfly provides a technical basis for the study of insect behaviour and a reference for tracking research. The proposed method was only suitable for a single moving insect, and the tracking trajectory may be incomplete when the target and background are similar. Further study is needed to apply this method detection and tracking to the high-speed flying of multiple targets and to improved tracking performance when the colour of the target and background are similar.

## Methods

### Insects

**Figure 8:**
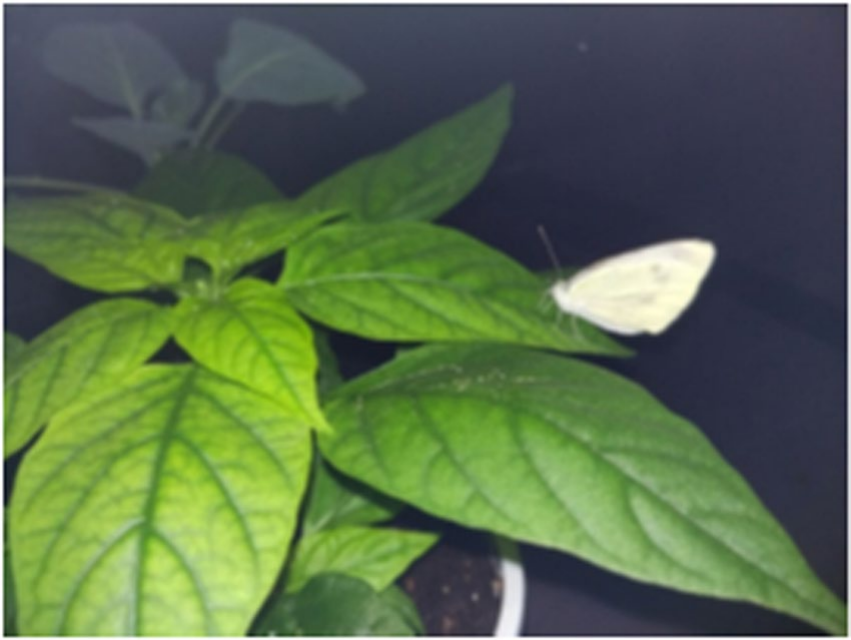
Cabbage butterfly used in the experiment.

The cabbage butterfly has a wide distribution worldwide, and cabbage butterfly adults are employed as the experimental subjects (Fig. 8); these adults have a grey-black body and white wings with fine scales. They prefer to fly under strong light in the daytime. A total of 26 cabbage butterfly adults were acquired from the Chinese Arid Area Research Institute of Water-Saving Agriculture, Northwest A & F University, Yangling, Shannxi, China from May 25 to June 8, 2016. Their body lengths were 12–20 mm and wing expanses were 45–55 mm. The captured cabbage butterflies were fed honey-water for 1–2 days before use in experiments.

### Experimental scene

Wind-tunnel technology is an important experimental setup to study the behaviour of insects^32^. This experiment was conducted in the wind tunnel of the Insect Behavioral Laboratory of Northwest A&F University. The dimensions of the wind tunnel are 1.8 m × 0.58 m × 0.58 m (L × W × H). Potted cabbage seedlings ~20 cm high and potted green pepper seedlings ~18 cm high were placed on the left side of the wind tunnel to serve as an experimental chemical source for the butterfly. An axial fan was installed on the right side of the tunnel to produce a weak airflow from right to left. In this paper, wind tunnel airflow velocity was set to 0.4 m/s to simulate the breeze in a natural environment.

### Design of the video capture system

**Figure 9:**
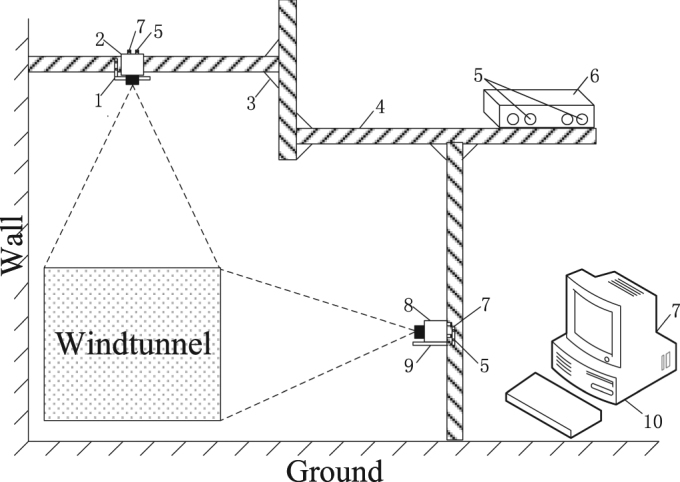
Video acquisition system. 1. Support plate 2. CCD1 3. Connecting pieces 4. Aluminium alloy extrusions 5. Trigger connecting port 6. Trigger 7. Net access 8. CCD2 9. Support plate 10. PC.

To obtain behavioural information from the butterfly, we designed an orthogonal binocular vision video acquisition system appropriate to the location and size of the wind tunnel (Fig. 9). The system consisted of two CCD cameras, a trigger, a computer, a camera-mounting bracket and ancillary equipment. To detect flight motion parameters for the fast-flying insects, we selected a gigabit network industrial camera (MICROVIEW, RS-A2300-GM60, BeiJing, China) with 1600*1200 pixels, a sensor of 7.2 × 5.4 mm, a lens (KOWA, LM3NCF, BeiJing, China) with a focal length of 3.5 mm, and a field of view of 89.0° × 73.8°. The camera was connected to a gigabit network card via an RJ45 interface to transmit grey video information. The optical axes of two cameras with same configuration were mounted orthogonally on brackets to obtain side-view and top-view image sequences, respectively. To effectively obtain 3D information for the targets, a MATLAB camera calibration toolbox was employed for internal parameter calibration of the two cameras^33,34^. To achieve two-camera synchronized shooting, an external trigger (MVM-B-0008-S00-V0001) was used to achieve synchronous acquisition. A 6-pin IO connector at the rear of the camera powered the camera and provided a trigger I/O. The trigger was connected to the two cameras via network cables connected to the computer. The unified trigger input pulse signals were set on the computer with control software to directly control the synchronized acquisition of two cameras.

### Data processing platform

The video data were obtained using the above system, and valid video segments were randomly selected for experimentation. Then, a computer with an Intel Core I5-2400 processor with 3.2 GHz dominant frequency, 8 GB memory and a 500 G byte hard drive, was used to process video data. All tests were conducted using the MATLAB 2014a environment.

### Cabbage butterfly target tracking

**Figure 10:**
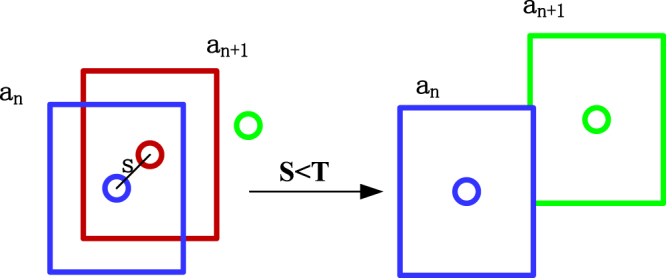
Schematic diagram for reacquiring the target. Circles represent the target positions. Green circles are the actual position of the target in the a_n+1_ frame, and the red circle is the wrong position when the target is lost. In this case, S < T, the target is redetected by BS, to obtain the correct target position in the a_n+1_ frame.

To obtain a 3D trajectory, it was first necessary to track the cabbage butterfly targets. In this study, the frame rates of the two cameras reached 60 fps to prevent the target displacement between frames from becoming too large when cabbage butterfly flying at maximum speed, and this improved the tracking accuracy. Henriques *et al*.^35^ proposed the KCF tracking algorithm in 2015, which is a new type of high-speed tracking algorithm. By constructing a classifier between the target and the background to determine the target, the achieved algorithm works at high speed with fast training and fast detection. Therefore, in applications to fast-moving target tracking, the KCF tracking algorithm has broad prospects. Tracking experiments using the KCF algorithm found that when the target was lost, the method was unable to re-detect and recognize the target. The background subtraction (BS) method is an effective motion detection algorithm, for which the motion area was detected by comparing the current frame with the background image difference^36^, and BS was used to regain the goal in this paper. Therefore, the KCF algorithm and BS method were combined in this paper to track cabbage butterfly targets. The principle of this method was to manually select the first frame target and to use the KCF-BS algorithm to achieve automatic target tracking. The main steps of the tracking algorithm for the cabbage butterfly target were as follows^35^:

1. Obtaining the cabbage butterfly base sample *a* and building a circulant matrix.
  In the original frames of the video, the rectangular area containing the butterfly and some surrounding environmental information were manually selected as the base sample *a*, which represents the characteristic information of the cabbage butterfly target. For the grey characteristic, with *a* as the two-dimensional matrix, the sample *a* was cyclically shifted to obtain the circulant matrix:
  1 $$A=[\begin{array}{ccccc}a & {R}^{1}a & {R}^{2}a & \cdots  & {R}^{n}a\\ {D}^{1}a & {D}^{1}{R}^{1}a & {D}^{1}{R}^{2}a & \cdots  & {D}^{1}{R}^{n}a\\ {D}^{2}a & {D}^{2}{R}^{1}a & {D}^{2}{R}^{2}a & \cdots  & {D}^{2}{R}^{n}a\\ \vdots  & \vdots  & \vdots  & \ddots  & \vdots \\ {D}^{n}a & {D}^{n}{R}^{1}a & {D}^{n}{R}^{2}a & \cdots  & {D}^{n}{R}^{n}a\end{array}]$$
  where *R*^*i*^ is the bias matrix circularly moving *i* places to the right; and *D*^*j*^ is the bias matrix circularly moving *j* places downward.
2. Diagonalization of the Fourier space of the circulant matrix and the search for a simplified linear regression function.
  The sample training process for butterfly target tracking was a ridge regression, which aimed to create a linear regression function *f*(*c*) = *w*^*T*^*c*, to make *f*(*a*_*i*_) = *w*^*T*^*a*_*i*_ = *b*_*i*_, and to find a *w* that minimizes errors in sample *a*_*i*_ and the desired output *b*_*i*_^37^:
  2 $$\mathop{{\rm{\min }}}\limits_{w}{\sum _{i}(f({a}_{i})-{b}_{i})}^{2}+\lambda {\Vert w\Vert }^{2}$$
  3 $$w={({A}^{H}A+\lambda I)}^{-1}{A}^{H}b$$
  where *w* is a regression coefficient; *λ* is a regularization parameter; *A*^*H*^ is the conjugate transpose of *A*; *I* is the unit matrix; and *b*_*i*_ is the desired output.
  The essential feature of a circulant matrix is the diagonalization with DFT, irrespective of the values generated^37^:
  4 $$A={F}^{H}diag(\hat{a})F$$
  where *F* is a discrete Fourier transform matrix; *F*^*H*^ is the conjugate transpose of *F*; $\hat{a}$ is the Fourier transform of *a*; and *diag* is the vector diagonalization.
  Substituting eq. (4) into eq. (3):
  5 $$\widehat{w}=\frac{\hat{a}\odot \widehat{b}}{{\hat{a}}^{\ast }\odot \hat{a}+\lambda }$$
  where $\hat{w}$ is the Fourier transform of *w*; and $\hat{b}$ is the Fourier transform of *b*.
3. Building the kernel ridge regression classifier
  Segmentation of the cabbage butterfly target from the background is a nonlinear problem, so it was transformed into a linear problem, and the solution was then determined. Here, a kernel function was used for the nonlinear transformation of the target tracking algorithm. A nonlinear mapping function *ψ*(*c*) transformed the sample characteristic space into a higher dimensional space so that the nonlinear regression became linearly separable^38^. Thus, the regression coefficient *w* and the regression function change to
  6 $$w=\sum _{i}{\alpha }_{i}\psi ({a}_{i})$$
  7 $$f(c)={w}^{T}\psi (c)={\alpha }^{T}\kappa (c)$$
  where *κ*(*c*) is a kernel function, *κ*(*c*) = *ψ*(*c*) **·** *ψ*(*a*_*i*_) mainly including Gaussian, polynomial and linear kernels; and *α* is a new regression parameter (the solution parameter is changed from *w* to *α*).
  From eq. (7), we can see that *f*(*c*) is a nonlinear function of *c*, but the linear function on *κ*(*c*) uses the optimization method of the linear function to solve *α*, with the result that:
  8 $$\alpha ={(K+\lambda I)}^{-1}b$$
  9 $$\hat{\alpha }=\frac{\hat{b}}{\hat{k}+\lambda }$$
  where *K* is the kernel function matrix (circulant matrix), *K*_*ij*_ = *κ*(*c*_*i*_, *a*_*j*_); and *k* is the first row of the constituent elements of *K*.
4. Fast-tracking of cabbage butterfly targets
  After designing a classifier, the next frame image was sampled in the predicted area and the sample was cyclically shifted to obtain a new circulant matrix as the sample set for detection. The sample with the largest response *f*(*c*_*i*_) was selected as the detected new butterfly target area, and the target displacement was judged from the relative displacement from the predicted area.
5. Re-detecting the target when it was lost When a target was lost, in the tracking process, the position of the tracking box was substantially unchanged (Fig. 2b1–d1). Thus, threshold T and distance S between the target centroid of the current frame and the target centroid of the previous frame were set for comparison to determine whether the target was lost. If S was less than T, the target was considered lost, and the new target centroid was obtained using the background subtraction method to serve as the target centroid of the current frame. The schematic diagram for reacquiring the target is shown in Fig. 10.

By repeating these steps frame-by-frame, we were able to achieve reliable target tracking. The time from frame acquisition to insect position was approximately 1.3 s.

### Acquisition of 3D trajectory parameters

The key to obtaining a 3D trajectory is to detect the coordinates of the target centroid of the butterfly. In this paper, two cameras were arranged orthogonally. Therefore, the 3-dimensional coordinates can be obtained only using the calibrated camera internal parameters and the orthogonal relationship between the two cameras. The coordinates *x*, *y* and *z* corresponded to the directions of the wind-tunnel length, depth and height, respectively. The top left of the wind tunnel was the coordinate origin. The parameters of the 3D trajectory were obtained as follows:

1. Side-view and top-view videos were tracked frame-by-frame using the algorithms described above. The centre coordinates of the rectangular box obtained were used as the two-dimensional centroid coordinates (*x*_*n*_, *z*_*n*_) and (*x*_*n*_, *y*_*n*_) of the butterfly in the corresponding side-view and top-view videos. The horizontal axis of the two image sequences was the same. There may be a deviation, but the average can be used instead of the abscissa, *y*_*n*_ corresponds to the *y* coordinate of 3D, and *z*_*n*_ corresponds to the *z* coordinate of 3D, so the 3D spatial coordinates are (*x*_*n*_, *y*_*n*_, *z*_*n*_).
2. The internal parameters of the cameras obtained during calibration were used to convert the 3D coordinates in pixel units to spatial coordinates in physical units, and the 3D coordinates of the target in each image frame were saved as.txt files for subsequent processing.
